# Impact of Mitophagy-Related Genes on the Diagnosis and Development of Esophageal Squamous Cell Carcinoma via Single-Cell RNA-seq Analysis and Machine Learning Algorithms

**DOI:** 10.4014/jmb.2407.07052

**Published:** 2024-09-23

**Authors:** Xuzhi Mo, Feng Ji, Jianguang Chen, Chengcheng Yi, Fang Wang

**Affiliations:** 1 Department of Thoracic Surgery, Dongying People's Hospital (Dongying Hospital of Shandong Provincial Hospital Group), Dongying 257088, P.R. China; 2 Department of Oncology, Dongying People's Hospital (Dongying Hospital of Shandong Provincial Hospital Group), Dongying 257088, P.R. China

**Keywords:** ESCC, mitophagy-related genes, prediction model, machine learning, single-cell RNA-seq

## Abstract

As a treatment for esophageal squamous cell carcinoma (ESCC), which is common and fatal, mitophagy is a conserved cellular mechanism that selectively removes damaged mitochondria and is crucial for cellular homeostasis. While tumor development and resistance to anticancer therapies are related to ESCC, their role in ESCC remains unclear. Here, we investigated the relationship between mitophagy-related genes (MRGs) and ESCC to provide novel insights into the role of mitophagy in ESCC prognosis and diagnosis prediction. First, we identified MRGs from the GeneCards database and examined them at both the single-cell and transcriptome levels. Key genes were selected and a prognostic model was constructed using least absolute shrinkage and selection operator analysis. External validation was performed using the GSE53624 dataset and Kaplan–Meier survival analysis was performed to identify *PYCARD* as a gene significantly associated with survival in ESCC. We then examined the effect of PYCARD on ESCC cell proliferation and migration and identified 169 MRGs at the single-cell and transcriptome levels, as well as the high-risk groups associated with cancer-related pathways. Thirteen key genes were selected for model construction via multiple machine learning algorithms. PYCARD, which is upregulated in patients with ESCC, was negatively correlated with prognosis and its knockdown inhibited ESCC cell proliferation and migration. Our ESCC prediction model based on mitophagy-related genes demonstrated promising results and provides more options for the management and clinical treatment of ESCC patients. Moreover, targeting or regulating PYCARD levels might offer new therapeutic strategies for ESCC patients in clinical settings.

## Introduction

Esophageal squamous cell carcinoma (ESCC) is characterized by high incidence and mortality rates [1]. It ranks seventh among the causes of cancer-related deaths worldwide [2], with more than half of these deaths occurring in China [3, 4]. The main therapeutic approach for ESCC comprises surgical removal of the tumor, along with the use of radiation and chemotherapy as additional treatments [5]. However, owing to vague initial symptoms, most cases are identified at a late stage, leading to a bleak outlook and a < 30% chance of survival for 5 years [6]. Therefore, there is an urgent need to explore the mechanisms underlying ESCC and improve treatment outcomes.

Mitochondria are vital organelles within cells that serve as the main reservoir of cellular energy and play pivotal roles in several cellular processes, including metabolism, proliferation, and differentiation [7, 8]. Accumulating evidence has linked mitochondria to cancer [9, 10]. Oxidative stress can induce apoptosis via mitochondrial pathways [11], and the excessive production of mitochondrial reactive oxygen species can trigger ferroptosis [12]. Mitophagy, the process of eliminating damaged or dysfunctional mitochondria, is vital for maintaining cellular homeostasis [13]. Dysregulated mitophagy may promote tumor development and confer resistance to anticancer therapies. For example, when Pink1/Parkin-mediated mitophagy is suppressed, the anti-cancer effects of quercetin against hepatocellular carcinoma become prominent [14]. TMBIM6 resists non-small cell lung cancer by inducing mitophagy [15]. However, the role of mitophagy-related genes (MRGs) in ESCC progression remains unclear.

In this study, given the close association between mitophagy and tumorigenesis, we employed a series of innovative bioinformatics methods to screen for genes related to mitophagy in ESCC. We then identified MRGs from the GeneCards databaseand used single-cell and transcriptome data to identify important MRG-associated genes using various machine learning algorithms, including random forest (RF) and support vector mchine (SVM). Additionally, we employed least absolute shrinkage, selection operator (LASSO), and Cox regression analyses to develop a predictive model for MRGs. Our model was externally validated to establish its reliability as a tool for estimating patient prognosis in clinical practice. Additionally, the model has potential utility for evaluating the tumor microenvironment, predicting immune therapy responses, and assessing tumor mutation burden, immune checkpoints, and drug sensitivity. For further cellular experiments, we used bioinformatic methods to identify *PYCARD* as the gene most significantly associated with survival in ESCC. By constructing a mitophagy-related prediction model at the single-cell level and exploring its relationship with ESCC, this study aimed to provide novel insightsinto the role of mitophagy in ESCC prognosis and diagnosis prediction.

## Materials and Methods

### Data Collection and Preprocessing

We downloaded transcriptome, clinical, and follow-up data on 93 patients with ESCC from The Cancer Genome Atlas (TCGA) database (https://portal.gdc.cancer.gov/) using TCGAbiolinks [16]. The transcriptome data were then normalized using Limma [17]. Additionally, we downloaded the ESCC-related datasets GSE161533 and GSE53624 from the Gene Expression Omnibus (GEO) database (https://www.ncbi.nlm.nih.gov/geo/) using GEOquery [18]. GSE161533 includes information about samples from 28 patients with ESCC and 28 matched normal tissues. After excluding samples with missing clinical data, GSE53624 contained transcriptome and clinical data from 111 patients with ESCC. All data were included in this study.

Finally, we obtained single-cell RNA sequencing data on six patients with ESCC, including those related to tumor and adjacent non-tumor tissues, from the GSE196756 dataset [19]. MRGs were obtained from the GeneCards database, and genes with a relevance score >1 were chosen (Table S1).

### Single-Cell RNA-seq Analysis and Data Processing

Seurat [20] was used to perform quality control on single-cell sequencing data from three patients in the GSE196756 dataset, filtering out cells with mitochondrial gene content >10% and low-quality cells (0 < nFeature_RNA < 3000). Using the Harmony package, batch effects were removed [21]. The FindClusters function was used to group cells with a resolution setting of 0.5, resulting in 19 clusters, which were subsequently visualized using the t-distributed stochastic neighbor-embedding (t-SNE) approach. The clusters were annotated into seven cell subtypes based on marker genes.

The activity of specific gene sets in each cell was then quantified using the AddModule Score function, and MRG scores were determined for each patient in the TCGA dataset using single-sample Gene Set Enrichment Analysis (ssGSEA). Finally, weighted correlation network analysis [22] was used to identify the gene clusters most significantly associated with the MRG scores. The TCGA-ESCC dataset was then used to compare gene expression between the cancer and non-cancer groups. This analysis was conducted using the Limma software, with criteria set at a minimum absolute log fold change of 1 and an adjusted *p*-value threshold of 0.05.

### Cell–Cell Communication Analysis

CellChat [23] was used to examine and deduce cell–cell communication networks in single-cell data. The examined interactions between cell groups were visualized with circle plots; important ligand-receptor interactions during signal transmission are displayed as heatmaps.

### Enrichment Analysis

Gene Ontology (GO) [24] was used to delineate gene activities, and the Kyoto Encyclopedia of Genes and Genomes (KEGG) [25] provided route maps depicting molecular interactions and reaction networks. GO and KEGG enrichment analyses were performed on core genes using the clusterProfiler package [26], with enrichment criteria defined as a *p*-value < 0.05 and a *q*-value < 0.05.

### Core Gene Screening Using Machine Learning

Two machine learning algorithms, SVM-RFE [27] and RF[28], were used to further screen these hub genes. Random Forest is an ensemble method based on decision trees, where each individual decision tree is trained on different datasets and randomly selected features. In our model, we set ntree=500 as the number of trees to be generated, and used the which.min function to find the point of minimum error (the optimal number of trees). SVM selects the optimal feature subset by recursively removing the least important features for classification. We set nfold=10 for ten-fold cross-validation in our model and identified feature genes based on accuracy and corresponding error rates. Finally, the genes selected by both models were intersected for further analysis.

### Prognostic Model Construction

LASSO can perform variable selection and complexity adjustment while fitting a generalized linear model, achieving calibration by minimizing the Bayesian Information Criterion [29]. We used the glmnet function to build the LASSO regression model, setting family = "binomial" and alpha=1 for iteration, with the maximum number of iterations set to 1000. Then, we used the cv.glmnet function for cross-validation to determine the optimal lambda, setting nfolds=10 for ten-fold cross-validation. LASSO regression was used to filter the prognostic markers and construct a prognostic model based on the prognostic coefficients. The risk score (RS) formula was as follows: RS = (Coef1Exp1) + (Coef2Exp2) + ... + (Coefi × Expi), where Coef represents the LASSO regression coefficients, with Exp representing the gene expression values, and i representing the number of genes.

Patients were categorized into high- and low-risk groups based on the median risk scores. The accuracy of the risk model was assessed using Kaplan–Meier and time-receiver operating characteristics (ROC) analyses. An AUC greater than 0.7 indicates good diagnostic performance. Univariate Cox regression analysis was used to examine the relationship between clinical variables (age, sex, stage, T stage, and N stage) and survival (*p* < 0.05). A nomogram was plotted for the multifactor model; the discrimination, accuracy, and clinical relevance of the model were evaluated using a receiver operating characteristic curve, calibration curves, and detrended correspondence analysis (DCA).

### Immune Analysis

We assessed the immune activity of tumors using the ESTIMATE method and TCGA-ESCC dataset [30]. This involved computing the immunological and stromal scores for each patient and comparing the variations in immune activity across the high- and low-risk groups. Immune cell invasion was then analyzed using CIBERSORTx [31] and further validated using ssGSEA [32]. We also analyzed common immune checkpoints owing to their crucial roles in cancer therapy.

### Mutation Analysis

To examine copy number variations in various risk categories, we obtained simple nucleotide variation and copy number variation data from 93 patients with ESCC from TCGA and used Maftools [33] to visualize the obtained results.

### Drug Sensitivity Analysis

We used pRRophetic [34] for personalized treatment to predict chemotherapy sensitivity in patients with ESCC with different MRG risk scores. By calculating the IC_50_ values for various anticancer drugs, we analyzed the correlation between risk scores and drug sensitivity, with *p*-values < 0.05 considered statistically significant.

### Cell Culture and siRNA Transfection

Five ESCC cell lines, including HEEC, KYSE510, KYSE150, KYSE30, and KYSE410, were obtained from the American Type Culture Collection (ATCC). The cells were grown in RPMI-1640 media (Kibbutz Beit HaEmek BI, Israel) containing 10% fetal bovine serum, at 37°C under 5% CO_2_. Passages did not exceed a maximum of 15. PYCARD RNA interference and negative control lentiviral vectors were used (GeneChem, China). The lentiviral vectors were transduced into KYSE150 and KYSE30 cells according to the manufacturer’s instructions.

### RNA Extraction and Quantitative Reverse Transcription Polymerase Chain Reaction

Cells were obtained via centrifugation, and RNA was isolated from ESCC cells using TRIzol reagent (Ambion, UK). SYBR Green Master Mix was used for qRT-PCR. The 2^-ΔΔCT^ method was employed to quantify the relative gene expression levels. The reference gene used was β-actin. The primers used were as follows: *PYCARD*: 5'-TGGATGCTCTGTACGGGAAG-3' (F) and 5'-CCAGGCTGGTGTGAAACTGAA-3' (R); β-Actin: 5'-GAGAAAATCTGGCACCACACC-3' (F) and 5'-GGATAGCACAGCCTGGATAGCAA-3' (R).

### Western Blotting

A ristocetin-induced platelet aggregation buffer (Beyotime, China) was used to extract proteins. The proteins were separated on a 10% sodium dodecyl sulfate-polyacrylamide gel and transferred onto a polyvinylidene difluoride membrane. The membrane was then blocked with a 5% skim milk solution and incubated with an appropriate primary antibody overnight at 4°C, followed by 2 h of incubation with the secondary antibody. The signals were detected, and optical density values were calculated using an advanced chemiluminescence kit (Amersham ECL Plus, Lumigen, USA). β-Actin was used as an internal reference.

### Transwell Assay

Matrigel (BD Biosciences, USA) was diluted at a ratio of 1:8 with serum-free RPMI1640 medium and added to each Transwell chamber at a volume of 50 μl. The chambers were incubated at 37°C for 1 h to allow the Matrigel to completely solidify. Subsequently, appropriately transfected cells were seeded into the upper chamber of the Transwell plate, while the lower chamber was filled with complete medium. After 24 h of incubation, cells on the upper surface of the membrane were removed using a cotton swab. The membranes were fixed in 95% ethanol for 15 min and stained with crystal violet. The cells that migrated through the membrane were counted using an optical microscope to evaluate their migratory capacity.

### Wound Healing Assay

The cells were placed in 6-well plates and grown until 90% coverage of the plate surface was achieved. A scratch was created using a pipette tip, and the area was washed thrice with phosphate-buffered saline and incubated in a serum-free medium for 24 h. Images of the wound area were then captured at 0 and 24 h using a microscope, and its size was quantified using the ImageJ software.

### Clone Formation Assay

ESCC cells in the exponential growth phase were seeded in 6-well plates and incubated for 12 days. Following the formation of colonies, the cells were treated with a 4% paraformaldehyde solution, stained with a 0.1% crystal violet solution, and the number of colonies was determined.

### Statistical Analysis

All data computations and statistical analyses were performed using the R software (version 4.1.3; R Foundation for Statistical Computing, Austria). Continuous variables are presented as mean ± SD. The Wilcoxon rank-sum test was used to compare differences between two groups. For comparisons involving more than two groups, the Kruskal–Wallis test was used. Unless otherwise specified, correlation analyses were conducted using Spearman’s method. Statistical significance was set at *p* < 0.05.

## Results

### Single-Cell Analysis and Functional Annotation of ESCC Samples

**Fig. 1:**
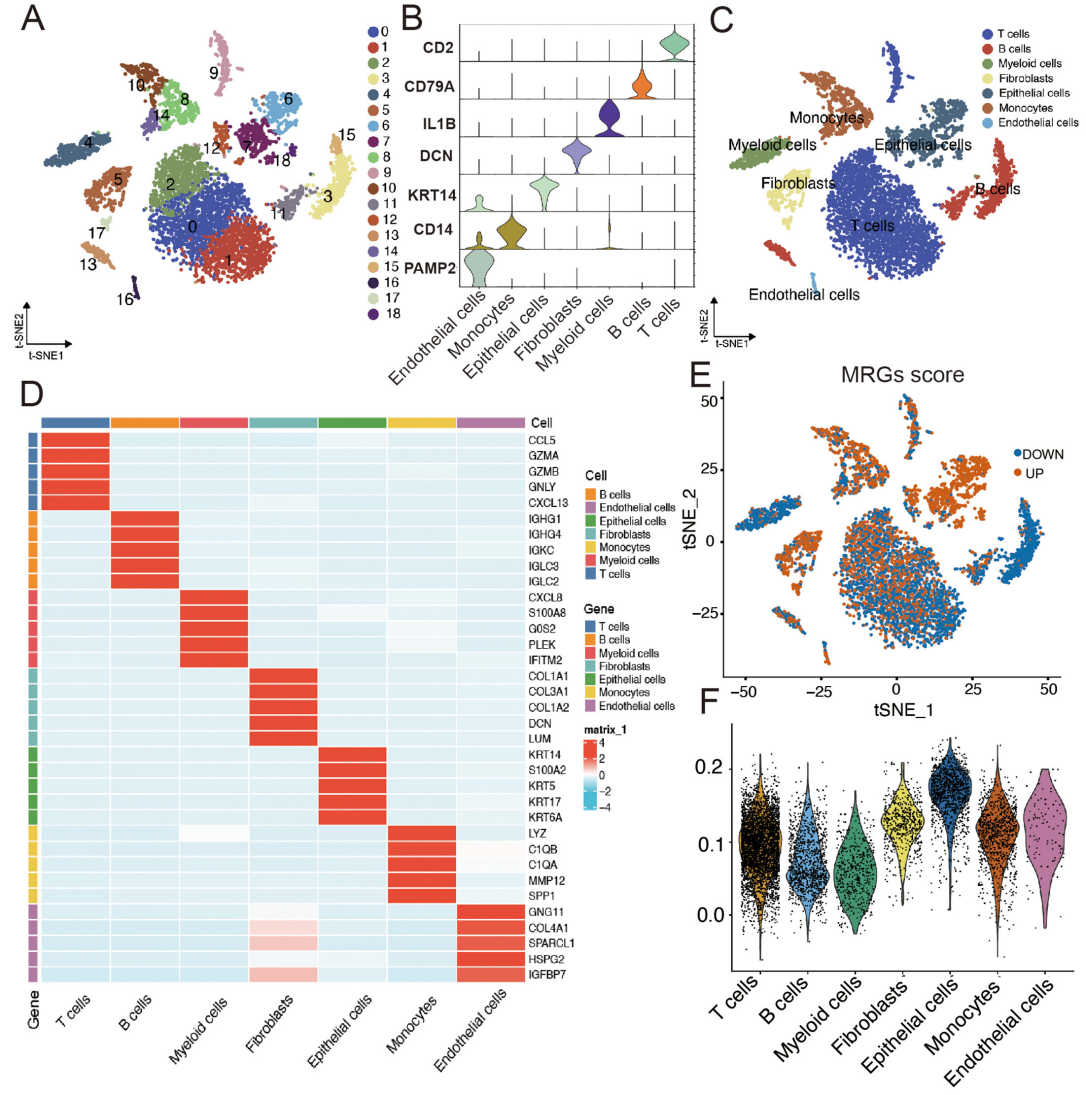
Characteristics of mitophagy-related genes in single-cell transcriptomics. (**A**) t-SNE plot showing 19 clusters of ESCC samples. (**B**) Marker genes for different cell types. (**C**) t-SNE plot showing cell types identified by marker genes. (**D**) Heatmap displaying the top 5 marker genes for each cell cluster. (**E**) Activity scores of mitophagy-related genes in each cell type. (**F**) Violin plot showing the expression of MRGs scores across different cell types.

After the t-SNE approach was employed to cluster single-cell ESCC samples into 19 distinct clusters (Fig. 1A), cells were categorized into seven primary clusters based on marker genes for various cell types, including T cells, B cells, myeloid cells, fibroblasts, epithelial cells, monocytes, and endothelial cells (Fig. 1B and 1C). A heatmap was used to display the top five marker genes for each cell type (Fig. 1D). To measure the activity of mitophagy-related gene sets in various cell types, the AddModuleScore function was used to compute expression levels. This involved categorizing the cells into two groups based on their MRG scores, *i.e.*, high- and low-score groups (Fig. 1E). Violin plots revealed that epithelial cells exhibited the highest MRG-related gene expression, followed by monocytes (Fig. 1F). Therefore, we selected epithelial cells for further analysis.

**Fig. 2:**
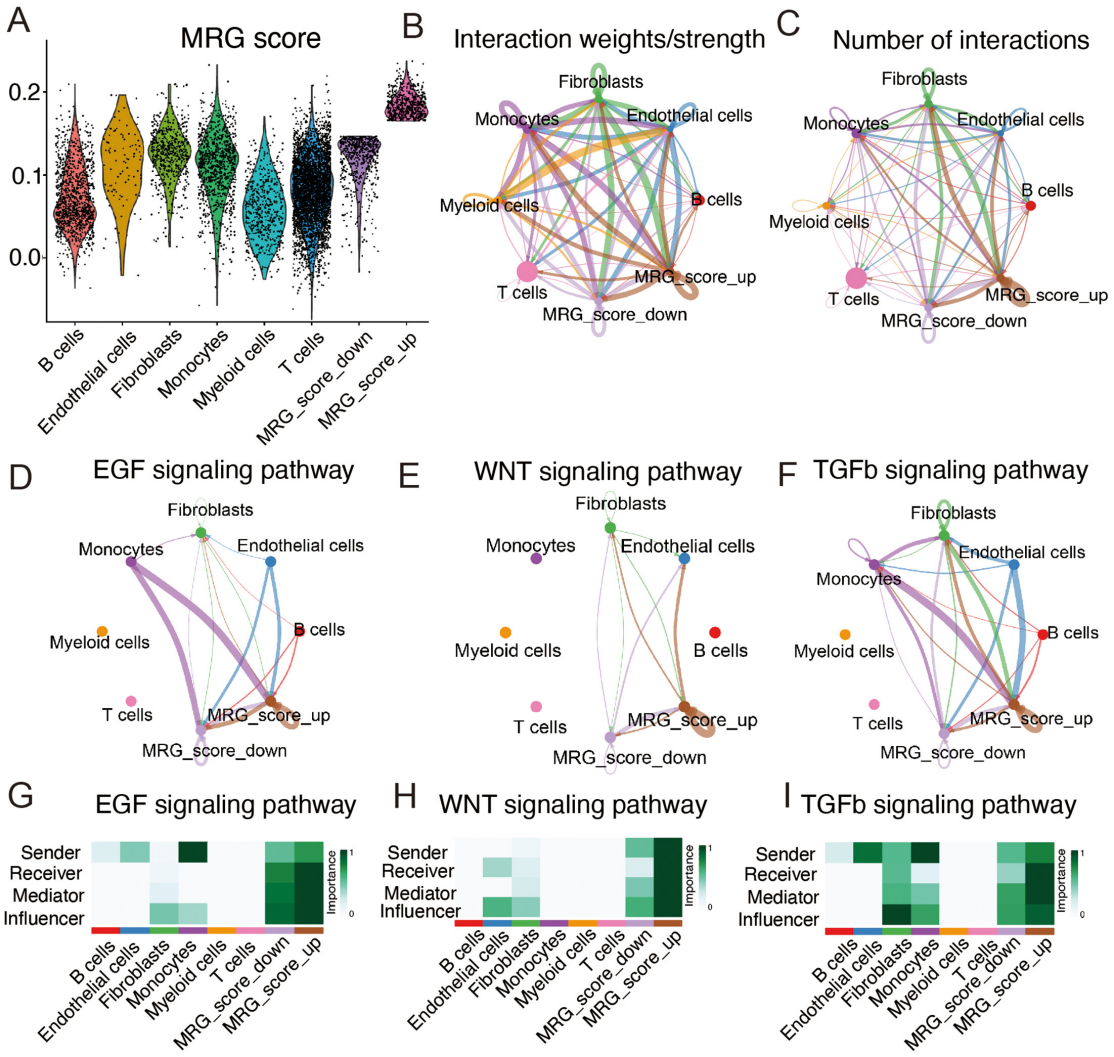
Further analysis of the correlation between MRGs scores and single-cell characteristics. (**A**) Epithelial cells divided into high and low MRG score groups based on median activity values. (**B, C**) Number and weight of interactions in the cell communication network. (**D-F**) Circos plots showing the expression in EGF, ENT, and TGFb cancer signaling pathways. (**G-I**) Heatmaps showing the roles of different cell types in pathway networks.

For further analysis, epithelial cells were split into high-and low MRG score groups based on the median expression activity (Fig. 2A). Assessment of cell-cell communication networks revealed more active interactions and higher interaction weights in the high-MRG score group than in the low-MRG score group (Fig. 2B and 2C). The high-MRG score group played significant roles in epidermal growth factor, WNT, and transforming growth factor-β (TGF-β) signaling pathways (Fig. 2D–2I), indicating their potential involvement in tumorigenesis and development. This suggests that mitophagy-related genes may be involved in the formation and progression of ESCC.

### Identification of MRG-Related Hub Modules and Genes Through Bulk RNA-seq

**Fig. 3:**
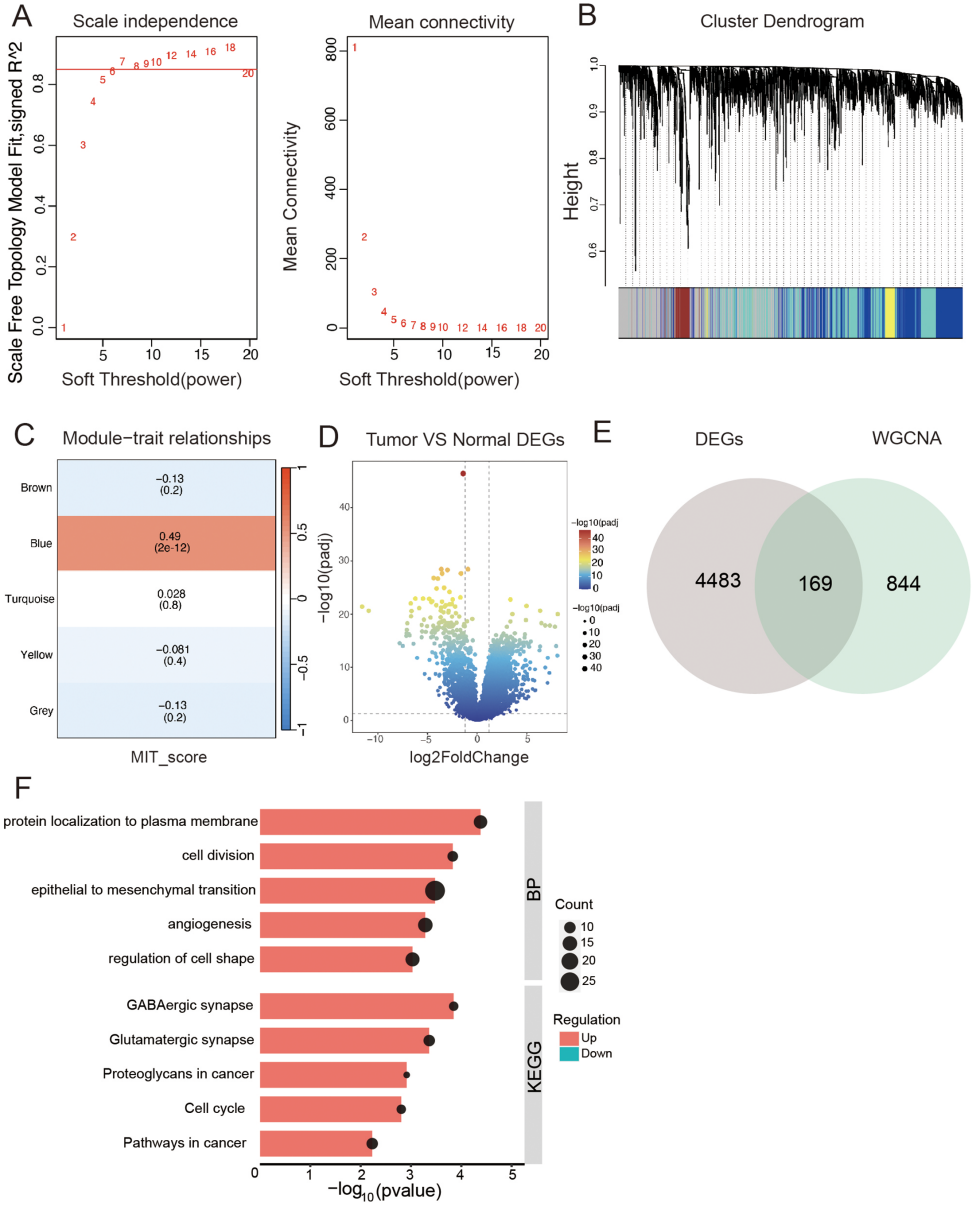
Identification of mitophagy-related genes. (**A, B**) MRGs scores for each sample calculated by ssGSEA algorithm for WGCNA analysis clustering. (**C**) Module-trait heatmap showing a strong correlation between the blue module and MRGs scores. (**D**) Volcano plot showing differential analysis results between tumor and normal samples in TCGA-ESCC. (**E**) Venn diagram showing the intersection of differentially expressed genes and genes in the blue module. (**F**) GO/KEGG enrichment analysis results for MRGs.

Values from the ssGSEA algorithm employed to calculate the activity scores of MRGs for each TCGA-ESCC sample were used as phenotypic data in the subsequent WGCNA analysis. To identify the modules most significantly associated with the MRG scores, a co-expression network was constructed using WGCNA (Fig. 3A and 3B), with an optimal soft threshold of 9 (R2 = 0.769), resulting in five modules (Fig. 3C). The Blue module was closely related to MRG scores (cor = 0.49), indicating that the genes within this module may have MRG-related functions. The TCGA-ESCC dataset was used to analyze differential expression between tumor and normal tissues. The analysis applied thresholds of |logFC| > 0.5 and adj.p < 0.05 to identify genes that were differentially expressed. These findings are presented in Fig. 3D. A Venn diagram intersected 1,013 genes in the blue module with differentially expressed genes, identifying 169 core genes (Fig. 3E). The identified genes showed enrichment in biological processes, including cell division, epithelial-to-mesenchymal transition, and angiogenesis. Additionally, they were associated with various KEGG pathways, including GABAergic synapses, glutamatergic synapses, cell cycle, and cancer pathways (Fig. 3F).

### Core Gene Selection and Prognostic Model Construction Using Multiple Machine Learning Algorithms

**Fig. 4:**
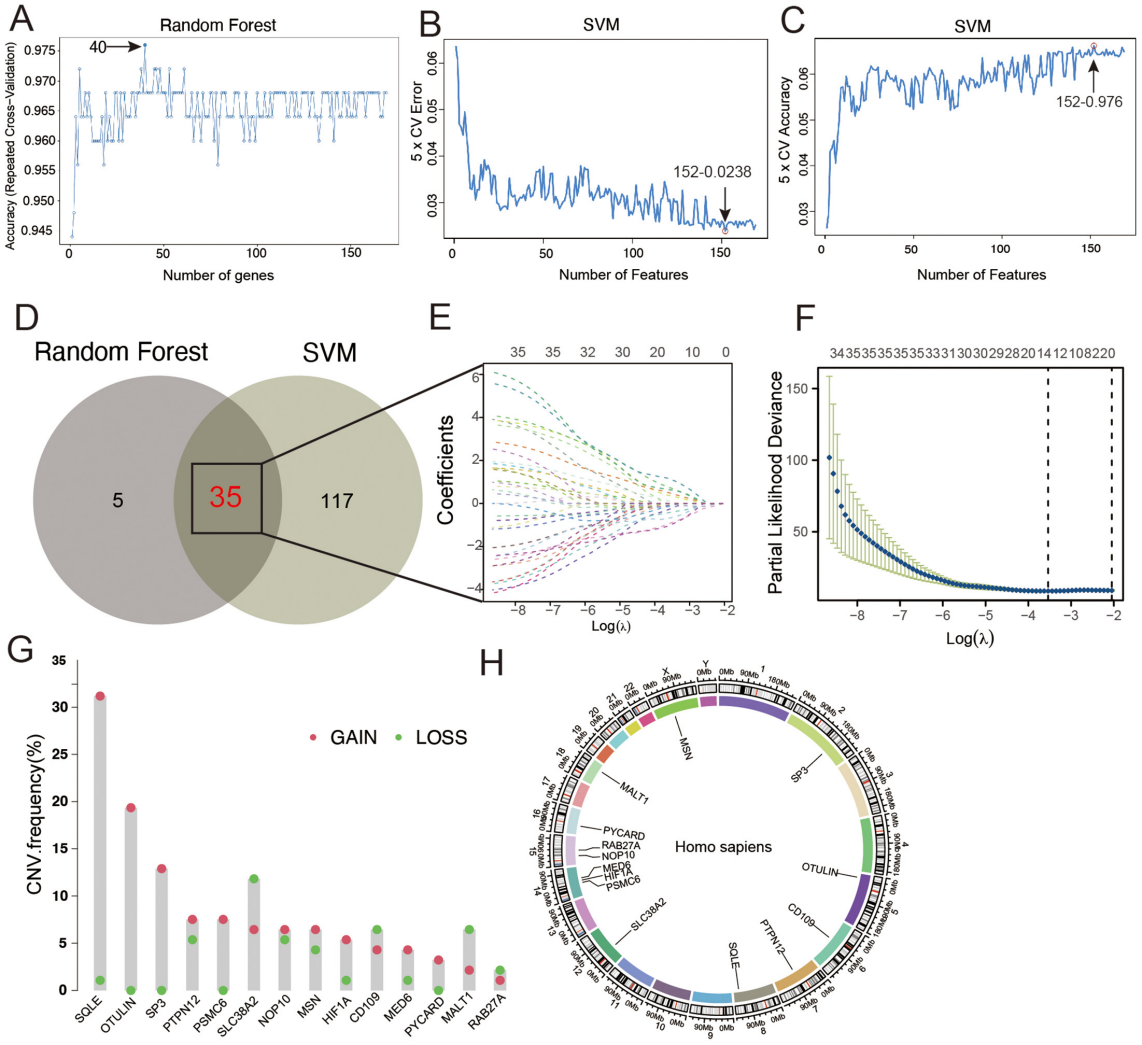
Screening of core genes using multiple machine learning algorithms. (**A-C**) Screening of core genes using Random Forest and SVM algorithms. (**D**) Venn diagram showing the intersection of genes from each machine learning algorithm. (**E, F**) LASSO regression analysis of the 13 screened genes. (**G, H**) CNV frequency distribution and chromosomal location information for core genes.

Random forest (Fig. 4A), SVM (Fig. 4B and 4C), and CoxBoost algorithms (Fig. 4D) were used to choose core genes from the 169 core genes, resulting in the selection of 13 genes after intersection in a Venn diagram (Fig. 4E). LASSO regression analysis was conducted (Fig. 4F and 4G) to obtain the regression coefficients for each gene. A risk score formula was subsequently created by multiplying the gene expression values by their respective coefficients and summing them; the patients were categorized into high- and low-risk groups according to the median risk score.

**Fig. 5:**
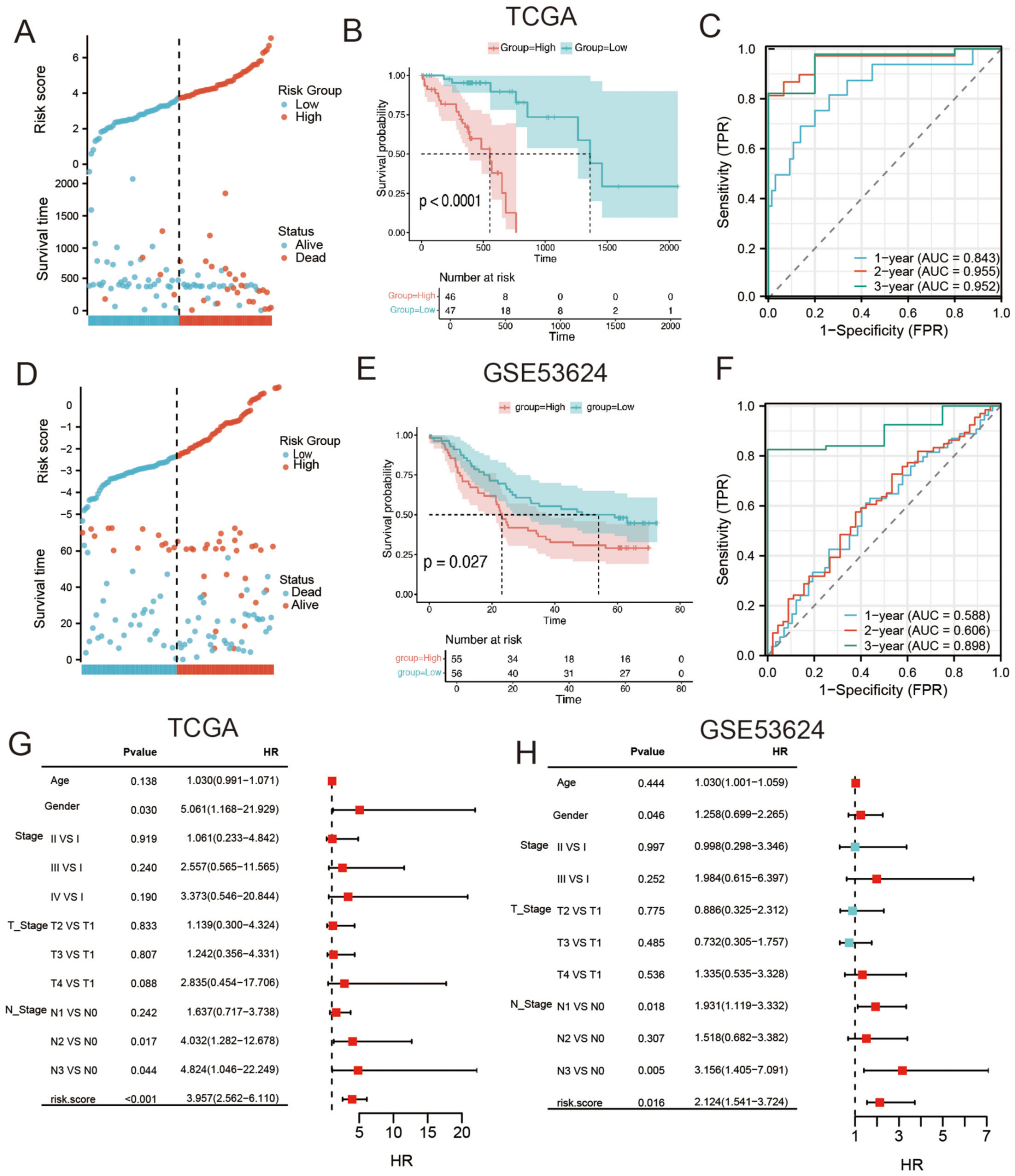
Construction and validation of the risk score. (**A**) In the TCGA dataset, deceased patients are mostly concentrated in the high-risk group. (**B**) K-M analysis shows lower survival in high-risk and low-risk groups. (**C**) ROC curve analysis indicates good predictive ability of the risk score for ESCC patients. (**D-F**) Further validation of risk factors, K-M survival, and ROC analysis in the external dataset GSE53624. (**G**) Univariate Cox regression analysis of Age, Gender, Stage, T Stage, N Stage, and risk score in the TCGA dataset. (**H**) Further validation in the external dataset GSE53624.

The risk factor plot shown in Fig. 5A indicates that deceased patients were predominantly concentrated in the high-risk group. Additionally, significant differences between the high- and low-risk groups were observed in the Kaplan–Meier analysis (Fig. 5B). A time-dependent ROC curve study showed that the risk score had a strong potential for risk prediction in patients with ESCC. The AUC values for 1, 2, and 3 years were 0.843, 0.955, and 0.952, respectively (Fig. 5C). Validation with the GSE53624 dataset confirmed higher mortality in high-risk patients (Fig. 5D) and significant differences in Kaplan–Meier (Fig. 5E) and ROC analyses, with AUCs of 0.588, 0.606, and 0.898 for 1, 2, and 3 years, respectively (Fig. 5F), indicating the high accuracy and reliability of the risk score. To investigate the impact of common clinical indicators on overall survival in ESCC, a univariate Cox regression analysis was performed on TCGA-ESCC data, analyzing factors such as age, sex, and tumor stage (Fig. 5G). This was validated in the GSE53624 dataset (Fig. 5H). Meanwhile, in the TCGA-ESCC datas*et al*so, there were significant differences between the high and low-risk score groups in terms of clinical characteristics (Fig. S1A–S1E). Furthermore, using the Kaplan–Meier survival curves, we observed that the risk scores showed a stable ability for predicting ESCC across various clinical characteristic strata (Fig. S1F–S1L).

**Fig. 6:**
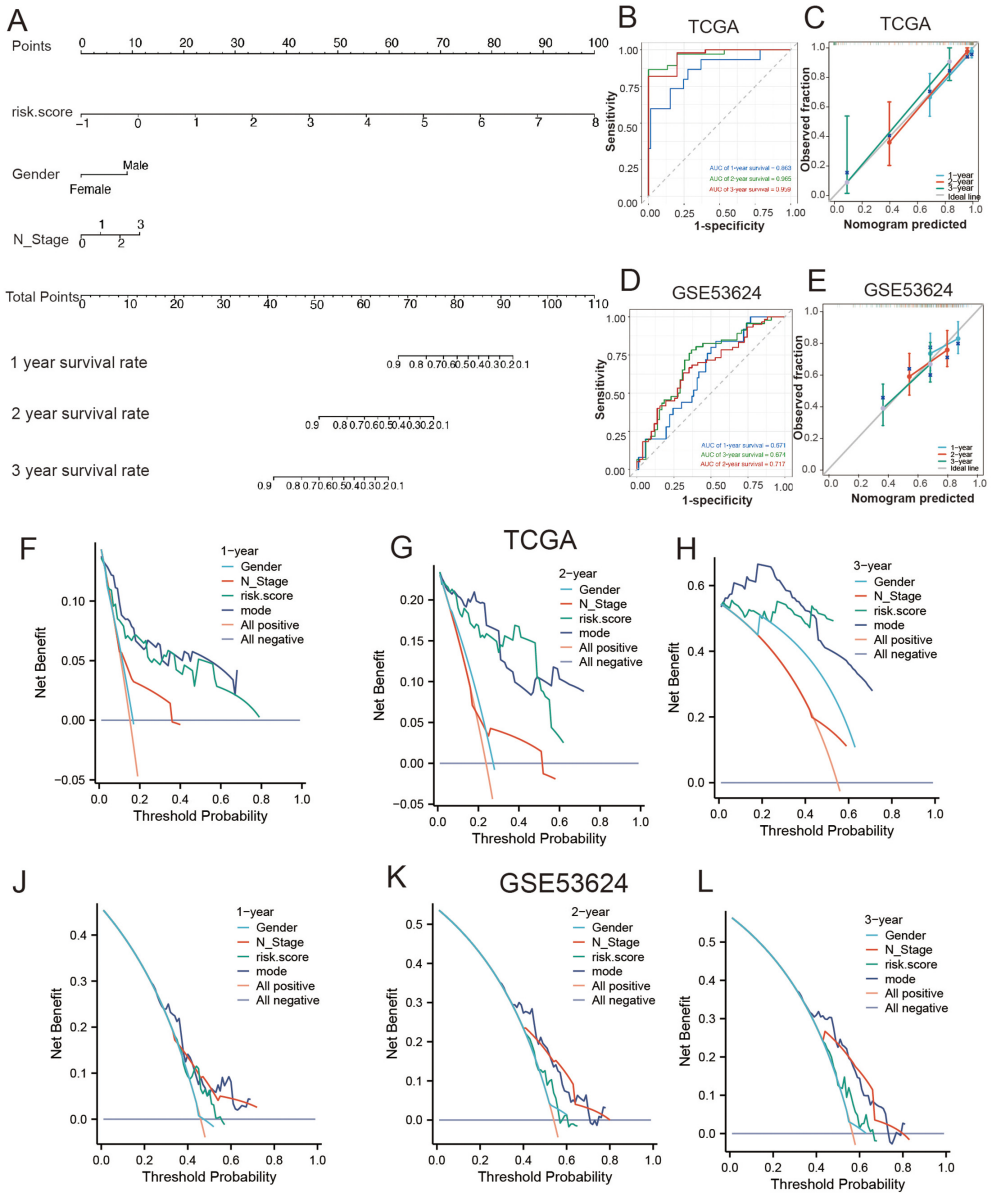
Construction and validation of the nomogram. (**A**) Construction of the nomogram based on risk score and clinical characteristics (Gender and N Stage). (**B, C**) ROC curve and Calibration analysis validation in the TCGA dataset. (**D, E**) Further validation in the external dataset GSE53624. (**F-H**) DCA analysis evaluating the clinical utility of the prognostic model at 1, 2, and 3 years in the TCGA dataset. (**J-L**) Further validation in the external dataset GSE53624.

Sex and N stage significantly affected survival; therefore, they were included in the nomogram (Fig. 6A). Model stability was validated using ROC curve analysis (Fig. 6B) and calibration analysis (Fig. 6C) in the TCGA dataset and further validated using the GSE53624 dataset (Fig. 6D and 6E). ROC analysis showed AUCs above 0.65 for 1–3 years, and calibration curves indicated consistent prediction trends with actual results, demonstrating the accuracy and stability of the nomogram. Finally, DCA was used to assess the clinical utility of the prognostic model for 1, 2, and 3 years in the TCGA dataset (Fig. 6F–6H); the results were validated with the GSE53624 dataset (Fig. 6J–6L), showing improved predictive performance over time.

### Immune Analysis

To investigate the correlation between risk scores and immunity, we performed an ESTIMATE analysis using the risk scores (Fig. S2A–S2C). The findings indicated a positive correlation between elevated risk scores and higher immune scores (*p* < 0.001) and ESTIMATES cores (*p* < 0.001). We used CIBERSORT to examine the infiltration of 22 distinct immune cell types across various groups (Fig. S2D). Macrophages M2 and monocytes showed higher levels of infiltration in the high-immune score group than in the low-immune score group. ssGSEA analysis yielded similar results (Fig. S2E). We further analyzed 14 immune checkpoints (Fig. S2F) and found that CD48, HAVCR2, IDO1, LAG3, LAIR1, SELPLG, and TIGIT were highly expressed in the high-score group, whereas CD200R1 was expressed at low levels. Moreover, we analyzed the relationship between risk ratings and the presence of immunological checkpoints (Fig. S2G).

### Genetic Variation and Drug Sensitivity Analysis

**Fig. 7:**
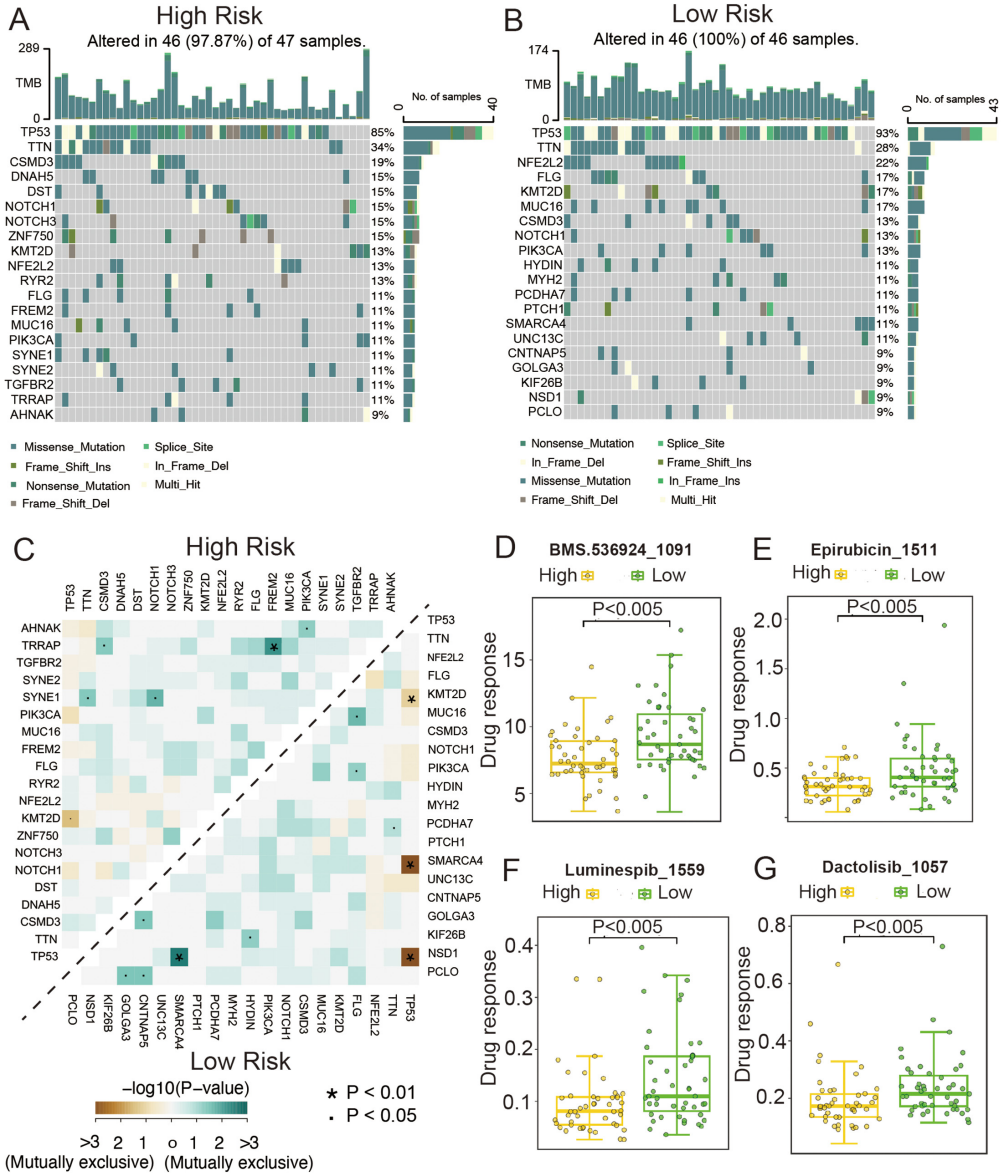
Gene mutation and drug sensitivity analysis between high and low-risk groups. (**A**) Waterfall plot of somatic mutations in high-risk patients in the TCGA-ESCC dataset. (**B**) Waterfall plot of somatic mutations in low-risk patients in the TCGA-ESCC dataset. (**C**) Heatmap showing the co-occurrence and exclusivity of the top 20 mutated genes in high and low-risk groups. (**D-G**) Analysis of drug sensitivity differences between high and low-risk groups using the GDSC database.

To investigate the differences in genomic mutations between the high- and low-score groups, we created waterfall plots for the high- and low-risk groups (Fig. 7A and 7B). TTN, TP53, and CSMD3 had higher mutation frequencies in both the groups. Additionally, we analyzed the co-occurrence and mutual exclusivity of the top 20 mutated genes in the high- and low-risk groups using heatmaps (Fig. 7C). To assess the sensitivity of patients with different risk scores to various drugs and small molecules, we downloaded the cell line mutation data and IC_50_ values of different anticancer drugs from the GDSC database. The results indicated significant differences in the IC_50_ values of several chemotherapeutic and small-molecule anticancer drugs between the high- and low-risk patients (*p* < 0.005), with BMS.536924_1091 (Fig. 7D), epirubicin _1511 (Fig. 7E), luminespib _1559 (Fig. 7F), and dactolisib _1057 (Fig. 7G) showing the most notable differences.

### PYCARD Promotes ESCC Cell Proliferation and Migration

**Fig. 8:**
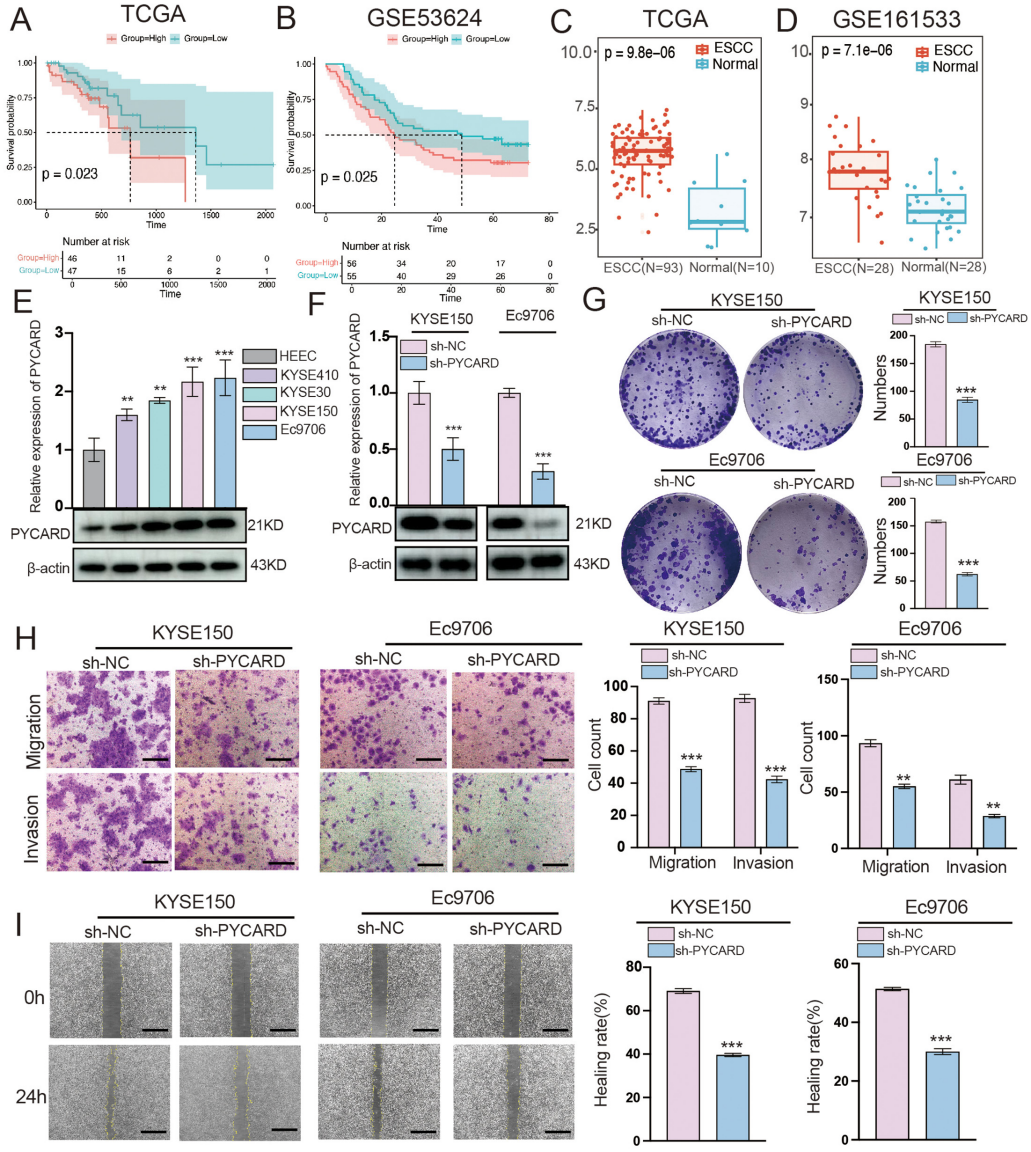
PYCARD promotes proliferation and migration of esophageal squamous cell carcinoma cells. (**A, B**) K-M survival analysis shows that patients with high PYCARD expression have lower survival rates in the TCGA and GSE53624 datasets. (**C, D**) High expression of PYCARD in ESCC patients in the TCGA and GSE161533 datasets. (**E**) qRT-PCR and Western blot analysis evaluating PYCARD expression in normal and ESCC cell lines. (**F**) Validation of PYCARD knockdown efficiency in KYSE150 and Ec9706 cells. (**G**) Effect of PYCARD on cell proliferation in KYSE150 and Eca9706 cells in colony formation assays. (**H**) Knockdown of PYCARD significantly inhibits the migration and invasion of ESCC cells compared to the control group. (**I**) Wound healing assays exploring the effect of PYCARD knockdown on the migration of KYSE150 and Eca9706 cells. *p < 0.05, **p < 0.01, ***p < 0.001. Scale bar: 100 μm.

To further study the impact of the 13 core genes on the survival of patients with ESCC, we performed a Kaplan–Meier survival analysis using the TCGA database and dataset GSE53624. PYCARD showed differential expression (Fig. 8A and 8B), with high expression indicating low survival rates. Transcriptomic analysis of TCGA (Fig. 8C) and GSE161533 data (Fig. 8D) revealed high PYCARD expression in patients with ESCC, suggesting its involvement in ESCC development. We also evaluated PYCARD protein expression in different cell lines (HEEC, KYSE410, KYSE30, KYSE150, and Ec9706) using qRT-PCR and western blotting. PYCARD was more highly expressed in ESCC cells than in HEEC, with the most significant differences observed in the KYSE150 and Ec9706 cell lines (Fig. 8E). We introduced lentiviral vectors to knockdown PYCARD in KYSE150 and Ec9706 cells to achieve a good knockdown efficiency (Fig. 8F). Colony formation, transwell migration, and wound healing experiments demonstrated that the suppression of PYCARD expression resulted in a notable decrease in the growth and movement of KYSE150 and Ec9706 cells (Fig. 8G–8I).

## Discussion

ESCC is a common and lethal gastrointestinal malignancy associated with various risk factors such as alcohol consumption, thermal injury, oxidative stress, and mitochondrial dysfunction [35]. The mechanisms underlying ESCC remain unclear, and there is a lack of early diagnostic markers and therapeutic strategies, which poses significant challenges for clinical treatment. Hence, it is imperative to identify novel therapeutic targets to improve ESCC treatment outcomes. Mitochondria play a crucial role in the development and progression of cancer [36-38], and mitophagy clears dysfunctional mitochondria, which is essential for cellular health. Aberrant mitophagy promotes the survival of damaged and mutated mitochondria, promoting malignancy [39]. Previous studies have shown that autophagy-deficient cancer cells exhibit increased metastasis [40, 41], which is closely associated with ESCC tumor growth [42] and poor prognosis [43]. However, the role of mitophagy in ESCC remains unclear.

The emergence of multiomics approaches has enhanced our understanding of disease mechanisms; these approaches have been widely used in cancer research to identify diagnostic or prognostic biomarkers and features, with significant results achieved for breast [44] and prostate cancers [45]. However, its application in ESCC is limited. This study utilized the AddModuleScore, ssGSEA, and WGCNA algorithms to detect genes associated with MRGs at both single-cell and bulk transcriptome levels. Our findings suggest various potential treatment options for ESCC. The risk score for mitophagy-related genes obtained using various machine learning methods to evaluate the essential genes was identified as an independent prognostic factor by univariate and multivariate Cox regression analyses in both TCGA-ESCC and independent datasets (GSE53624). We developed a nomogram using a multivariate analysis. Kaplan–Meier survival, ROC, calibration curve, and DCA analyses demonstrated that this nomogram effectively estimated patient prognosis. Therefore, this tool can help physicians create customized treatment strategies that are directly linked to the specific risk level of each patient. Overall, it enhances clinical outcomes and has the potential to enhance overall survival rates.

In this study, we identified 13 core genes from the 169 intersecting genes to construct a risk score. Further analysis of these genes revealed that PYCARD was highly expressed in ESCC and had prognostic value; additionally, its knockdown significantly inhibited the proliferation and migration of ESCC cells in vitro. Therefore, PYCARD was considered a significant MRG in our risk-prediction model.

Located on chromosome 16, PYCARD is a simple adapter protein that recruits pro-caspase-1 to inflammasomes [46] and plays a critical role in inflammation and apoptosis regulation [47], particularly in immune response regulation [48, 49]. Mounting data suggest that PYCARD plays a crucial role in the development and advancement of tumors [50]; for example, in pancreatic adenocarcinoma, 90% of tumor samples show elevated PYCARD expression when compared with adjacent normal tissues, correlating with a poor prognosis [51]. This is the first study to propose that PYCARD expression has a biological function in ESCC.

Notably, the cell communication analysis of single-cell transcriptomes revealed different communication patterns between tumor cells with high and low MRG scores. Cells with high MRG scores were crucial in the epidermal growth factor, WNT, and TGF-β signaling pathways, suggesting that these cells may regulate ESCC progression through these pathways. These findings are important for understanding the development of ESCC and may provide clues about targets for therapeutic interventions.

The tumor microenvironment is crucial for cancer recurrence and spread [52, 53] and has a major impact on the effectiveness of immunotherapy and chemotherapy [54]. Our study found significant immune differences between patients with high and low MRG scores; those with high MRG scores had higher proportions of neutrophils and M2 macrophages than those with low scores. Previous studies have shown that neutrophils promote angiogenesis through vascular endothelial growth factor and other pro-angiogenic factors that are crucial for tumor development [55]. M2 macrophage polarization is closely linked to ESCC progression [56, 57] and radiotherapy resistance in locally advanced ESCC [58], which is consistent with our finding that patients with high MRG scores have poorer prognoses than those with low scores. Drug sensitivity analysis revealed that the patients with high MRG scores had greater sensitivity to chemotherapy, especially to BMS.536924, epirubicin, luminespib, and dactolisib, than those with low scores, providing new insights into systemic treatment strategies for ESCC involving mitophagy.

Despite its significant findings, our study had certain limitations. First, although our bioinformatics results were validated using both the training set and an external independent validation set, further verification using RNA sequencing results is needed to bolster our conclusions. Second, since we only explored esophageal squamous cell carcinoma in our study, the general applicability of these findings to other cancers needs further confirmation. Third, large-scale multicenter prospective studies are still needed to confirm our findings before they can be applied in future clinical practice.

## Conclusion

Using multiomics techniques, we constructed a predictive model based on mitophagy-related genes from single-cell and bulk transcriptomics and conducted an in-depth analysis of prognosis, immune infiltration, genetic mutations, and drug sensitivity. This study offers valuable tools for estimating the prognosis and preventing and tailoring medical treatments for patients with ESCC, providing novel insights into the molecular pathways involved in ESCC formation and progression. Subsequent investigations showed elevated PYCARD expression in ESCC, which facilitates cell growth, movement, and infiltration. These findings indicate that PYCARD is a promising target for therapeutic interventions in ESCC. In summary, our study elucidates further the relationship between mitophagy and ESCC, paving the way for future research in this area.

## Supplemental Materials

Supplementary data for this paper are available on-line only at http://jmb.or.kr.
