# Supplementary material for: Impact of Mitophagy-Related Genes on the Diagnosis and Development of Esophageal Squamous Cell Carcinoma via Single-Cell RNA-seq Analysis and Machine Learning Algorithms
Source: J Microbiol Biotechnol. 2024 Sep 23;34(11):2362–75. doi: 10.4014/jmb.2407.07052 (PMC11637838; doi:10.4014/jmb.2407.07052)
Supplement: Supplementary file 1 [file jmb-34-11-2362-supple.pdf]

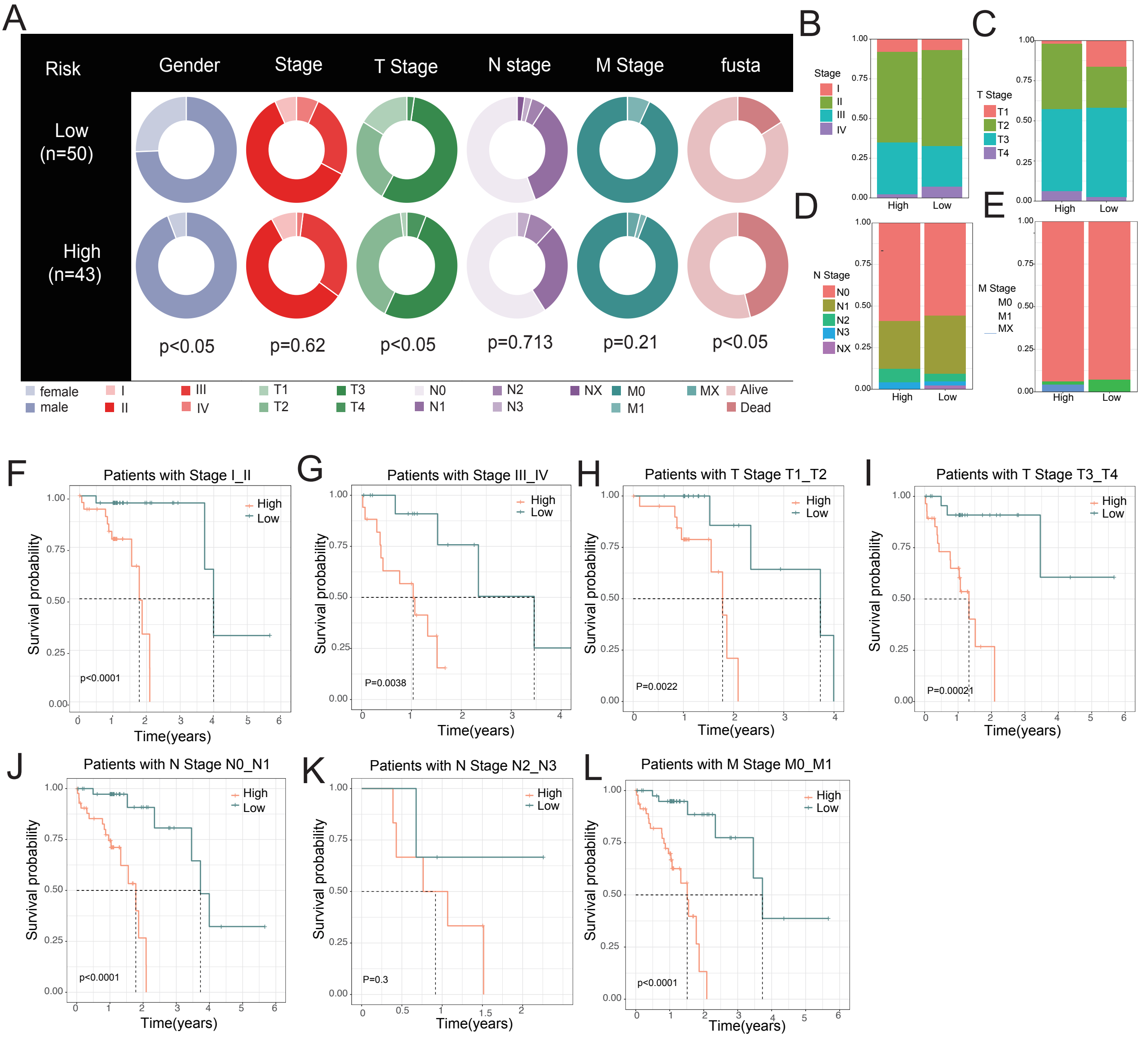

A
Risk
Gender
Stage
T Stage
N stage
M Stage
fusta
Low (n=50)
High (n=43)
p<0.05
p=0.62
p<0.05
p=0.713
p=0.21
p<0.05
female
male
I
II
III
IV
T1
T2
T3
T4
N0
N1
N2
N3
NX
M0
M1
MX
Alive
Dead
B
Stage
I
II
III
IV
High
Low
C
T Stage
T1
T2
T3
T4
High
Low
D
N Stage
N0
N1
N2
N3
NX
High
Low
E
M Stage
M0
M1
MX
High
Low
F
Patients with Stage I_II
p<0.0001
G
Patients with Stage III_IV
P=0.0038
H
Patients with T Stage T1_T2
P=0.0022
I
Patients with T Stage T3_T4
P=0.00021
J
Patients with N Stage N0_N1
p<0.0001
K
Patients with N Stage N2_N3
P=0.3
L
Patients with M Stage M0_M1
p<0.0001
Survival probability
Time(years)
High
Low

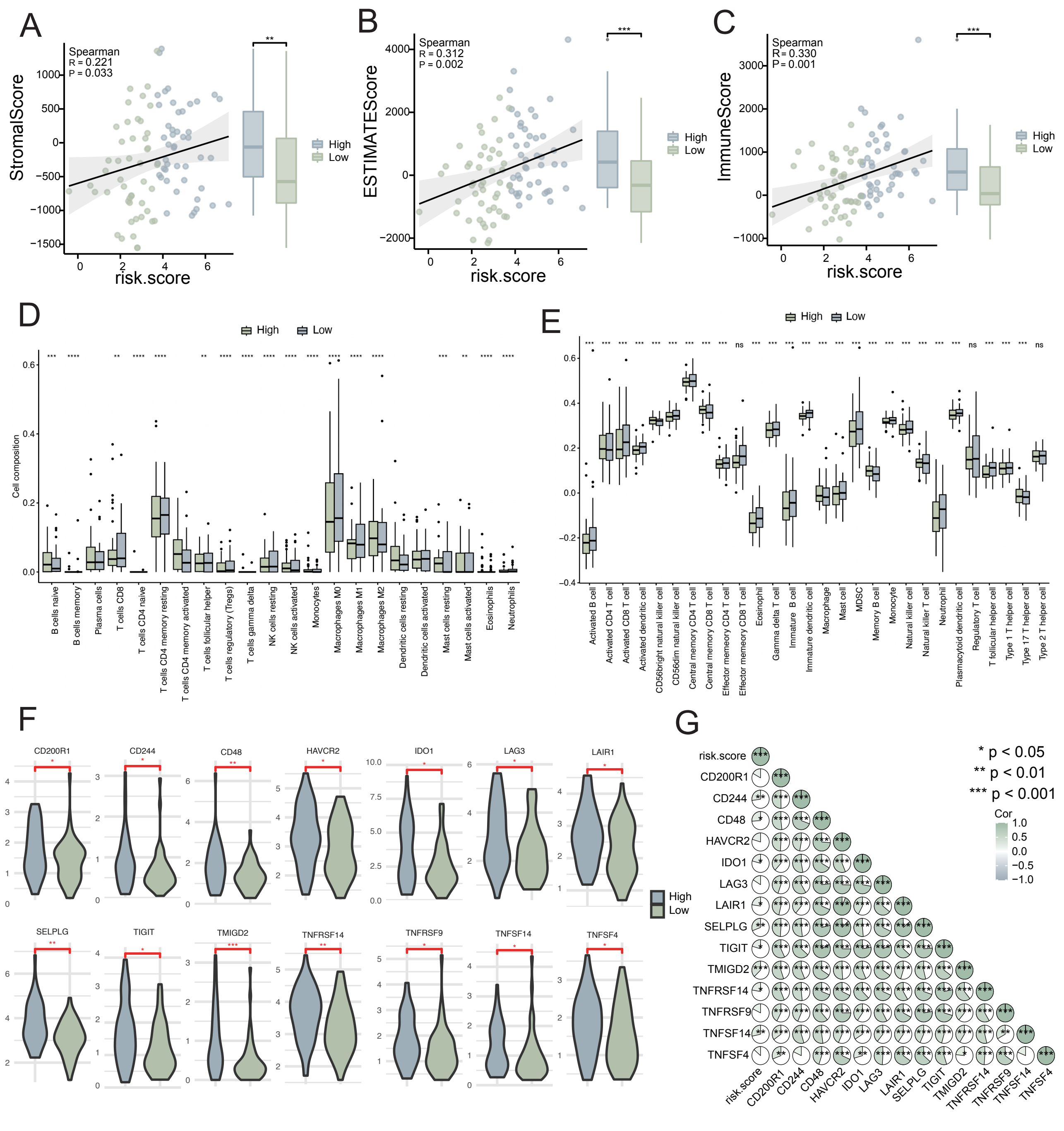
A
Spearman
R = 0.221
P = 0.033
StromalScore
risk.score
High
Low
B
Spearman
R = 0.312
P = 0.002
ESTIMATEScore
risk.score
High
Low
C
Spearman
R = 0.330
P = 0.001
ImmuneScore
risk.score
High
Low
D
High
Low
Cell composition
B cells naive
B cells memory
Plasma cells
T cells CD8
T cells CD4 naive
T cells CD4 memory resting
T cells CD4 memory activated
T cells follicular helper
T cells regulatory (Tregs)
T cells gamma delta
NK cells resting
NK cells activated
Monocytes
Macrophages M0
Macrophages M1
Macrophages M2
Dendritic cells resting
Dendritic cells activated
Mast cells resting
Mast cells activated
Eosinophils
Neutrophils
E
High
Low
Activated B cell
Activated CD4 T cell
Activated CD8 T cell
Activated dendritic cell
CD56bright natural killer cell
CD56dim natural killer cell
Central memory CD4 T cell
Central memory CD8 T cell
Effector memeory CD4 T cell
Effector memeory CD8 T cell
Eosinophil
Gamma delta T cell
Immature B cell
Immature dendritic cell
Macrophage
Mast cell
MDSC
Memory B cell
Monocyte
Natural killer cell
Natural killer T cell
Neutrophil
Plasmacytoid dendritic cell
Regulatory T cell
T follicular helper cell
Type 1 T helper cell
Type 17 T helper cell
Type 2 T helper cell
F
CD200R1
CD244
CD48
HAVCR2
IDO1
LAG3
LAIR1
SELPLG
TIGIT
TMIGD2
TNFRSF14
TNFRSF9
TNFSF14
TNFSF4
High
Low
G
risk.score
CD200R1
CD244
CD48
HAVCR2
IDO1
LAG3
LAIR1
SELPLG
TIGIT
TMIGD2
TNFRSF14
TNFRSF9
TNFSF14
TNFSF4
* p < 0.05
** p < 0.01
*** p < 0.001
Cor

Table S1. The mitophagy related genes extracted from GeneCards database classified by the relevance score

Relevance score	Count	Gene symbol
> 5	15	PRKN PINK1 MAP1LC3B VDAC1 FUNDC1 MFN2 SQSTM1 MAP1LC3A PHB2 ATG13 AMBRA1 ULK1 UBC TOMM20 USP30
4-5	10	CLEC16A DNM1L RIMOC1 UBA52 MFN1 OPTN HUWE1 PGAM5 SLC25A4 RNF41
3-4	29	ATG7 UBB SLC25A5 VPS13D TOMM40 VPS13C ATG4D TAFAZZIN ATG4B USP15 GABARAPL2 TOMM7 BNIP3L BECN1 ABCE1 CERS1 ATG5 TOMM70 BNIP3 TIGAR RHOT1 RPS27A LRBA ARFIP2 TOMM22 PHB1 FBXO7 RAB7A SPATA33
2-3	37	MAP1LC3B2 MUL1 GABARAP CHUK ATG9A TP53 CISD1 FKBP8 CALCOCO2 CNOT4 CSNK2A1 TBK1 VDAC2 CSNK2A2 MTX1 ATG4A BCL2L13 SNCA MON1A PELO HDAC6 LRRK2 PRKAA1 MTARC2 PTRH2 BCL2L1 FIS1OPA1 ATG14 TDRKH GBA1 ATG12 UBE2L3 MFF MAP1LC3C PI4KB SIRT1
1-2	181	WIPI1 VCPNBR1 SPATA18 HTRA2 SESN2 ATG4C SAMM50 IMMT MAPK14 FANCC TBC1D5 STX10 STK4 NLRP3 ATAD3AMARCHF5 PLSCR1 BCL2 SRCCSNK2BTOMM5 MTERF3 TOMM6 MIEF1 BAG5 PEX13 MTX2 HSPA8 OGT RETREG1 MAVS PTEN CHCHD3 GABARAPL1 PARL TSC2 VDAC3 HK2MAPK1 TSPO MAP2K1 FOXO3 MDH1 TBC1D15 OCIAD1 USP8 PI4K2A STOM KRT15 BLOC1S1 HIF1A ATM LRPPRC ATP1B1 APP BCAS3 PHAF1 ZFYVE16 BET1 GDAP1 WDR26 MCL1 LINC-PINT NR4A1 ALB HSPD1 HTTATG16L1 TFEB NIPSNAP1 BAG6 CDC37 SIRT3 MST1 TRAP1 ATG2B TGFB1 NFE2L2 UQCRC2NIPSNAP2 TFE3 VAPA DSP CAV1 TOM1 NME4 USP36 UBXN1 RMC1 RNF31 TCHP MTOR GPD2 SHC1 USP33 STX17 RNF121 CANX EPHA2 SLC12A6 ITCH SLC12A4 STEAP3 FADS2 UBE2G2 VPS35 ANO6 EMC1 LPCAT3 ARL6IP5 MON2 AMFR AKAP1 REEP5 SNX3 GDI2 CHMP2A ESYT1 ATG2A SPG21 RMDN3 SEC22B ARMCX3 TBC1D17 CCZ1B FLOT2 SH3GLB1 SREBF2 MIR155 PPARGC1A ALDH2 HSPA1L HMGB1 MAP2K3 LMAN1 FBXW7 ARIH2

	ATP5IF1 MAP2K2 TIMM23 UBXN6 FTMT HSPA9<br>WIPI2 ZFYVE1 TIMM44 HRAS TFRC NDUFA10<br>NRF1 KRAS PRKAA2 AKT1 RCAN1 RB1CC1<br>USP35 TUFM PRPF8 BAX MAPK15 VIM<br>MIR106B MIR93 MIR25 HK1IGF1 NDUFV1<br>OSBPL5 RDH13 TAX1BP1 CKAP4 MRPS2 PRKCD<br>BIRC2 HSPA1A TXN HNRNPM STOML2 ATAD3B<br>LOC105378097
